# Evaluating EEG complexity and spectral signatures in Alzheimer’s disease and frontotemporal dementia: evidence for rostrocaudal asymmetry

**DOI:** 10.1038/s41514-025-00243-y

**Published:** 2025-06-09

**Authors:** Kassra Ghassemkhani, Kevin S. Saroka, Blake T. Dotta

**Affiliations:** 1 Behavioural Neuroscience, Laurentian University, Sudbury, ON Canada; 2 Biology Programs, School of Natural Science, Laurentian University, Sudbury, ON Canada

**Keywords:** Biomarkers, Ageing

## Abstract

Accurate classification of neurodegenerative disorders remains a challenge in neuroscience. Using open-source electroencephalography (EEG) data, we investigated electrophysiological signatures to differentiate frontotemporal dementia (FTD) from Alzheimer’s disease (AD) via complexity measures. Traditional relative band power analysis showed consistent increases in lower-frequency activity but did not distinguish the two disorders after correction. In contrast, fractal dimension and long-range temporal correlations (LRTCs) revealed distinct topographical differences: AD exhibited rostral dominance in fractal dimension, whereas FTD showed caudal dominance. Both disorders demonstrated reduced LRTCs, particularly in caudal regions, indicating disrupted large-scale neural dynamics. These findings suggest that complexity-based EEG features may offer a reliable, cost-effective tool for distinguishing neurodegenerative conditions, complementing traditional neuroimaging approaches.

## Introduction

The diagnosis of neurodegenerative disorders typically relies on neuroimaging techniques such as positron emission tomography (PET), magnetic resonance imaging (MRI), or a combination of both^1–3^. Their high spatial resolution enables the identification of biomarkers associated with neurodegenerative disorders and holds potential for differential diagnosis. However, the high cost of MRI and PET remains a significant limitation. Electroencephalography (EEG) is a cost-effective, non-invasive technique that measures electric potentials that are generated by large populations of neurons across the scalp. Where EEG lacks spatial resolution, it has an advantage over MRI and PET in that it can measure within the operating speed of neurons (millisecond range), whereas MRI and PET measure in the range of seconds^4^. Despite the broad range of recording of EEG, fluctuations in amplitude and frequency have been shown to be consistent across multiple scales, from microelectrodes to intracranial and scalp recordings^5^. Thus, scalp EEG recordings are viable for reflecting small-scale neuronal dynamics. This is particularly relevant to neurodegenerative disorders, where neuronal dynamics are significantly altered. Moreover, EEG’s millisecond temporal resolution enables the detection of features that PET and MRI cannot capture.

Given the ability of EEG to capture neuronal dynamics at a fine temporal scale, power spectral analysis provides key insights into neurodegenerative disorders. Band power can be computed as absolute band power or as power relative to the entire spectrum, and alterations in these measures have been observed in neurodegenerative conditions. In the resting state, the alpha rhythm (8–13 Hz) is dominant, characterized by large amplitudes, and is often linked to cyclic inhibition and cortical excitability^4,6–9^. In Alzheimer’s disease (AD) and frontotemporal dementia (FTD), alpha band power is consistently lower than controls^10^. Conversely, slower frequencies such as delta (1–4 Hz) and theta (4–8 Hz) show significant increases in individuals with mild cognitive impairment^11^. These spectral shifts may reflect disturbances in the excitation-inhibition balance, a key feature of neurodegenerative pathology^12^. Given that inhibitory processes are closely tied to alpha activity, reductions in alpha power could indicate disruptions in inhibitory tone, potentially contributing to cognitive decline. While amyloid beta deposits and hyperphosphorylated tau pathology are hallmarks of AD and FTD, their exact impact on EEG spectral features remains an area of active investigation. Emerging evidence suggests that EEG-based measures could extend beyond traditional band power analysis to capture more nuanced disruptions in network dynamics, offering potential for improved biomarker development in neurodegenerative disorders. A technique growing in popularity is the observation of slow to fast band ratios. For instance, the frontal theta/beta ratio has seen great usage in assessing cognitive function and in individuals with attention-deficit/hyperactivity disorder^13–15^. The findings indicate that a higher theta/beta ratio is associated with lower attentional capacity, as measured by the Conners Continuous Performance Test (CPT), suggesting that this ratio could serve as a reliable biomarker for attentional deficits in ADHD populations^13^. Additionally, in AD and Lewy body disease, positive correlations have been observed between the theta/alpha ratio and theta/beta ratio with the various symptoms of both disorders, with the presence of theta/beta ratio in various regions being able to differentiate between the two disorders^16^. The theta/beta ratio has been among the most studied power ratios in EEG literature; the observation of other band pairings can possibly lend more insights on neurodegenerative disorders.

Another advantage of EEG is the wide range of signal processing methods available to researchers. In addition to band power analysis, a common approach for identifying EEG biomarkers is microstate analysis, which captures short-duration (60–120 ms) changes in global field power^17^. In AD, alterations in canonical microstate D, a distinct EEG pattern defined by polarity differences between the front (rostral) and back (caudal) regions of the scalp, have been observed. Specifically, the duration and frequency of this state are significantly reduced, and its spatial distribution is altered compared to controls^18^. These changes suggest a disruption in large-scale network coordination, particularly within the frontoparietal attention network, which is implicated in executive function deficits in AD^19^. Beyond microstate analysis, functional connectivity measures, such as coherence, provide additional insights into disrupted network dynamics in AD. Coherence, which assesses phase and amplitude similarities between signals from different brain regions, allows for the observation of interactions between neural structures rather than isolated activity. Notably, AD patients exhibit reduced alpha-band coherence across caudal regions, particularly within temporal, parietal, and occipital sensor pairings, indicating a weakening of long-range cortical connectivity^20^.

Signal complexity measures are less commonly used than frequency- and power-based EEG analysis techniques in the study of neurodegenerative disorders. These measures assess variations in both the spatial and temporal dynamics of neuronal activity, providing insights beyond conventional spectral analyses. One such measure is fractal dimension, traditionally computed using a time-series box-counting technique to quantify the space-filling properties of a signal^21^. While box-counting methods have been widely used to estimate signal complexity in EEG, more recent approaches such as the Higuchi Fractal Dimension (HFD) provide improved resolution of multiscale signal dynamics. HFD is particularly well-suited to EEG, as it does not rely on arbitrary segmentation and better accommodates the temporal structure of neural signals. Previous research has shown the utility of the HFD method^21–27^. In the context of AD, reductions in fractal dimension, particularly in parietal regions during resting-state EEG, have been interpreted as a loss of signal complexity in caudal cortical areas^22,23^.

Another common complexity measure is long-range temporal correlations (LRTCs), which assess the persistence of fluctuations in EEG amplitude over extended timescales. While techniques such as band power, microstate analysis, and coherence primarily capture transient (seconds) dynamics, LRTCs reveal how neuronal activity is structured over longer periods (minutes)^28^. In healthy brain networks, EEG signals exhibit scaling behavior, where fluctuations in amplitude follow power-law distributions across multiple timescales^28^. However, disruptions in this scaling behavior have been reported in AD, with evidence suggesting a breakdown in long-range temporal dependencies, particularly in posterior cortical regions^29^. These findings align with broader principles of complexity loss in neurodegenerative disorders, where brain networks exhibit reduced adaptability and diminished hierarchical organization. As such, LRTC and fractal dimension analyses offer promising avenues for capturing global signal breakdown in AD, potentially serving as biomarkers for disease progression.

Integrating multiple EEG analysis techniques provides a comprehensive approach to identifying signal abnormalities in neurodegenerative disorders. Historically, distinguishing between different forms of dementia based on broad electrophysiological effects has been challenging. However, advancements in EEG methodologies offer the potential to enhance differential diagnosis. To assess the generalizability of our findings, we also conducted a post hoc validation using a second open-access EEG dataset. This dataset, which included a larger number of probable AD patients but fewer age-matched controls, provided an opportunity to test whether our observed spectral and complexity-based EEG features were consistent across independent samples. In this study, we analyze global brain activity, as well as rostral and caudal sensor data, using both band power and signal complexity measures. Our primary objective is to determine whether EEG-based metrics can reliably differentiate between FTD and AD in comparison to age-matched controls. Additionally, we aim to assess EEG’s utility as a scalable approach for identifying electrophysiological signatures associated with neurodegeneration.

## Results

### Relative band power differences between groups

**Fig. 1:**
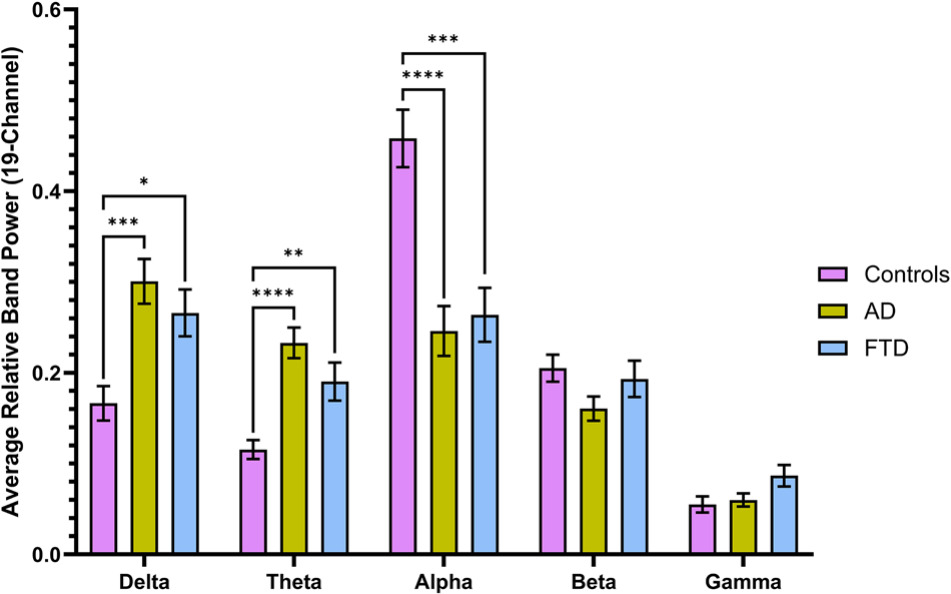
Comparison of the 19-channel average of relative power values across 5 frequency bands: delta (1–4 Hz), theta (4–8 Hz), alpha (8–13 Hz), beta (13–30 Hz), and gamma (30–45 Hz). Significant differences are noted with *, **, *** and **** (p < 0.05, p < 0.01, p < 0.001, and p < 0.0001, respectively). Error bars represent standard error of the mean.

**Fig. 2:**
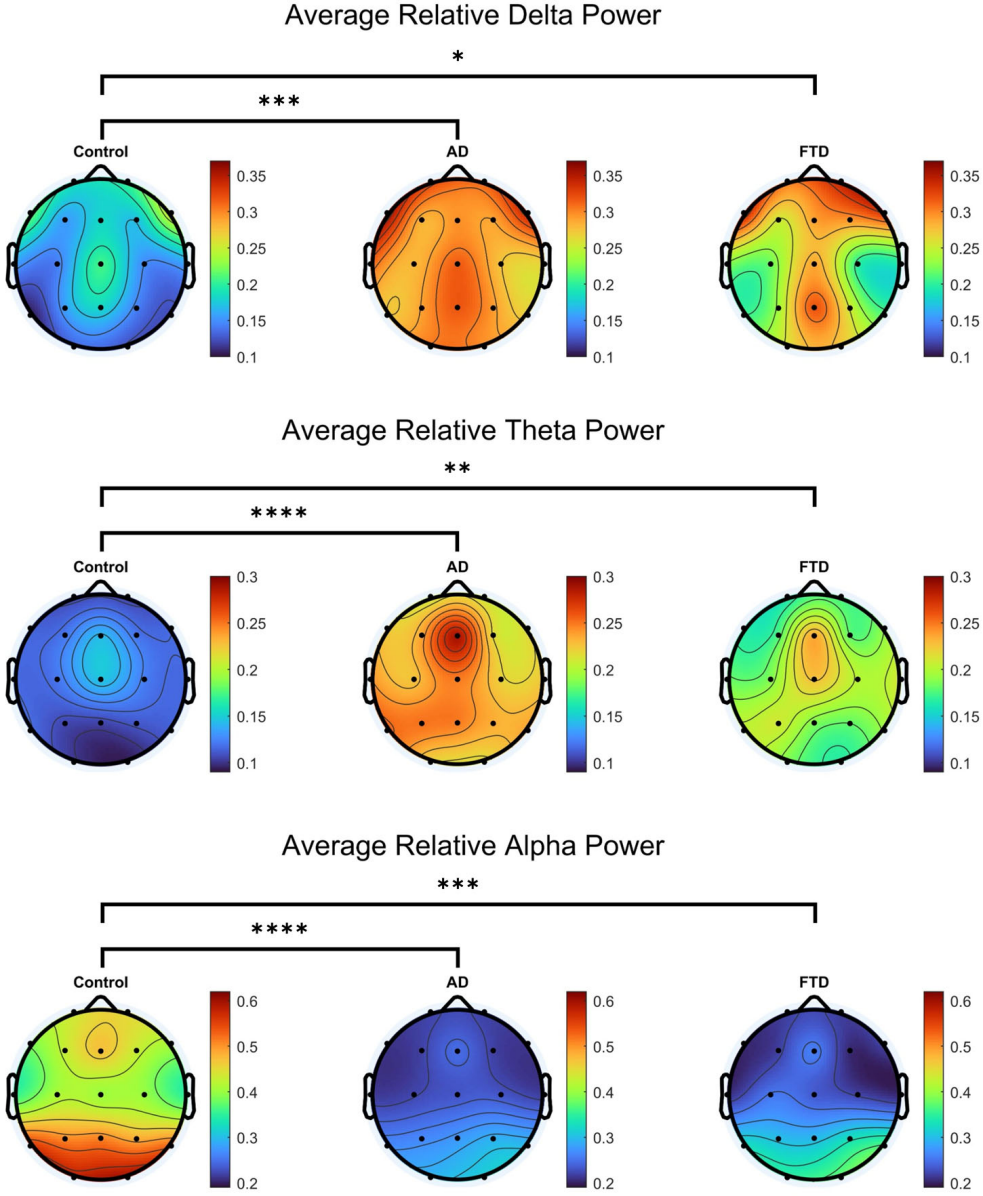
Topographic plots of the average relative power values for delta, theta, and alpha frequency bands. Black dots represent each of the 19 channels and are arranged according to the 10–20 system. Significant differences are noted with *, **, ***, and **** (p < 0.05, p < 0.01, p < 0.001, and p < 0.0001, respectively).

A multivariate analysis of variance revealed significant differences between groups when loading the 5 frequency bands [Pillai’s Trace = 0.423, F(8, 164) = 5.504, p < 0.0001] (Figs. 1 and 2). Post-hoc comparisons using Tukey’s HSD confirmed that when AD was compared to controls, significantly higher values were observed in relative delta [t(63) = 4.156, p = 0.0002, Cohen’s d = 1.0580] and theta power [t(63) = 5.577, p < 0.0001, Cohen’s d = 1.4309], with lower values observed in relative alpha [t(63) = 5.080, p < 0.0001, Cohen’s d = 1.2648]. Additionally, when comparing FTD to controls, similar increases were observed in delta [t(49) = 3.188, p = 0.0191, Cohen’s d = 0.8893] and theta [t(49) = 3.430, p = 0.0097, Cohen’s d = 0.9337], alongside a significant decrease in relative alpha power [t(49) = 4.350, p = 0.0002, Cohen’s d = 1.2466]. No significant differences in beta and gamma power were observed between groups after controlling for multiple comparisons.

**Fig. 3:**
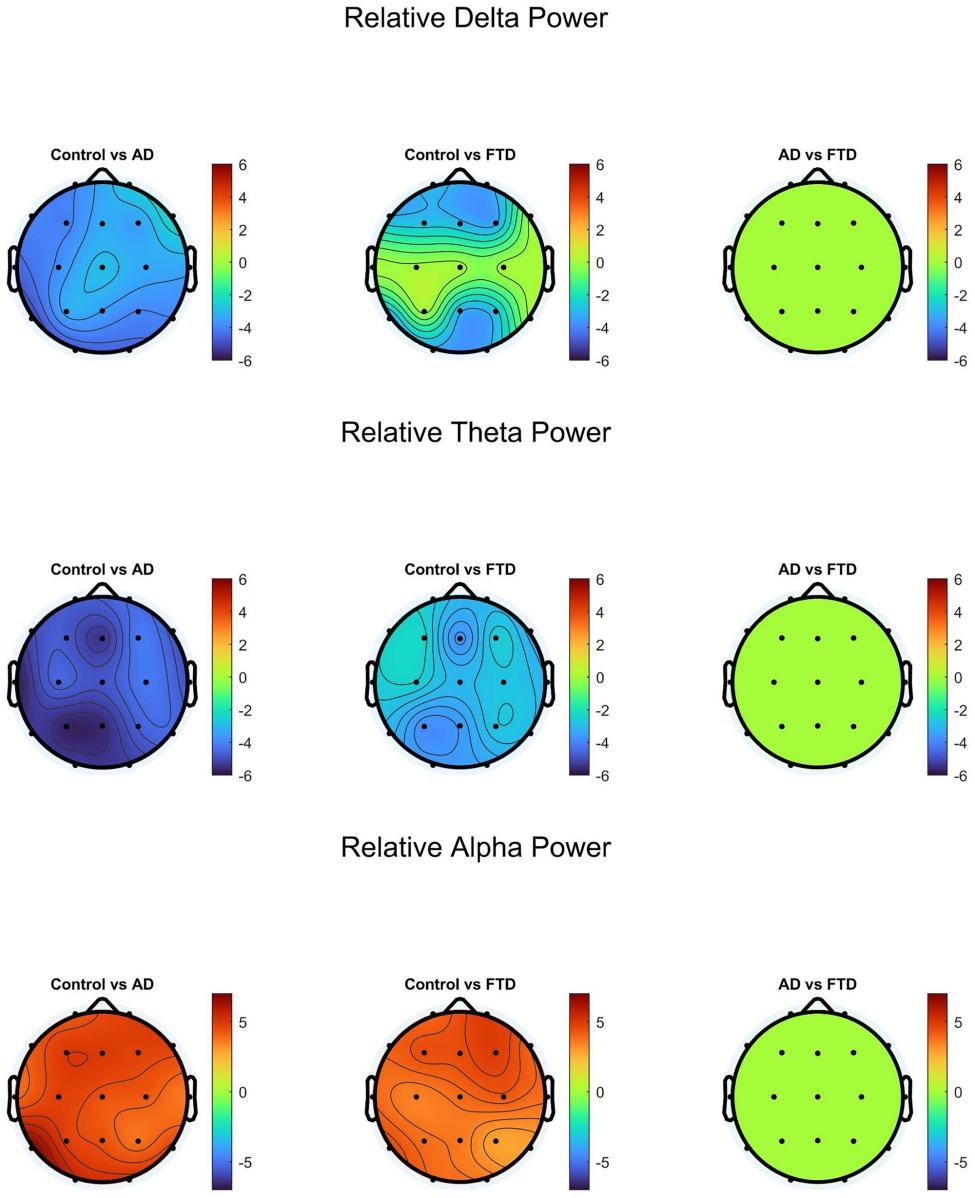
Topographic plots of the t-value comparisons with channels that passed the False discovery rate (FDR) correction in relative power values for delta, theta, and alpha frequency bands. Black dots represent each of the 19 channels and are arranged according to the 10–20 system. Color bars represent the t-values. The leftmost column compares controls to AD, the middle column compares controls to FTD, and the rightmost column compares AD to FTD.

To visualize the spatial distribution of observed power differences, we generated per-channel topographic t-value maps for delta, theta, and alpha bands displaying the significant differences that passed the false discovery rate (FDR) correction (Fig. 3). These significance plots revealed that the largest group differences were localized over posterior and temporal regions, particularly in comparisons between AD and controls, and to a lesser extent, FTD and controls. No consistent spatial patterns emerged in the AD versus FTD comparison, supporting the notion that spectral features alone do not reliably differentiate the two disorders.

### Fractal dimension and rostrocaudal differences between AD and FTD

**Fig. 4:**
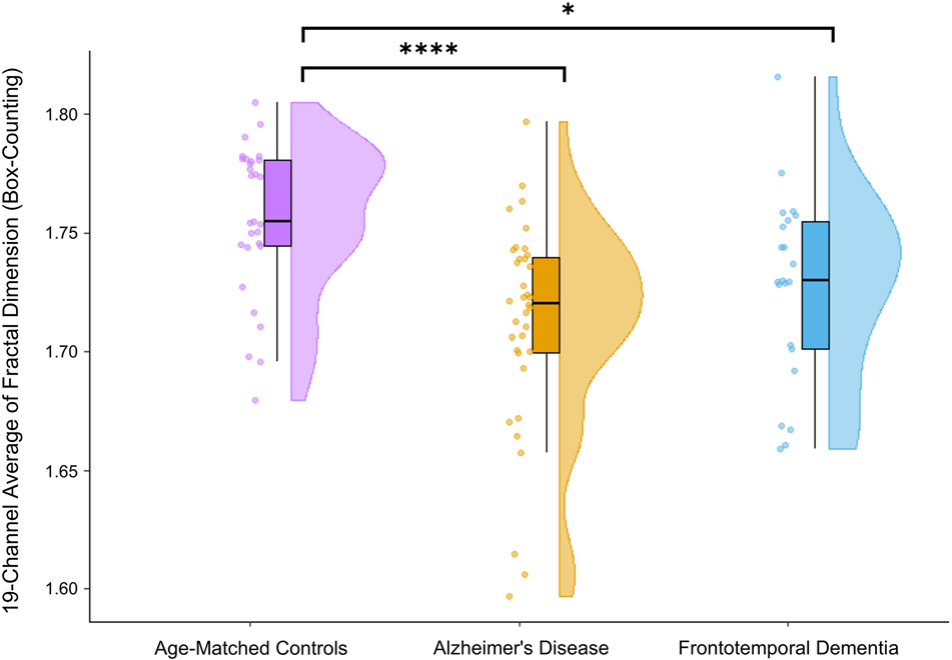
Raincloud plot comparing the 19-channel mean fractal dimension values between AD, FTD, and age-matched controls. Significant differences are noted with * and **** (p < 0.05 and p < 0.0001, respectively). On the left of each entry are the raw data points. In the middle is a boxplot which includes the interquartile range, and the data point range represented by vertical lines above and below the interquartile range. On the right is the point density plot represented as a half violin.

A one-way ANOVA revealed a significant effect of diagnosis on global (19-channel) broadband fractal dimension values [F(3, 87) = 9.966, p = 0.0001, r² = 0.1918]. Post-hoc comparisons using Tukey’s HSD indicated that fractal dimension values were significantly lower in the AD group compared to age-matched controls [t(63) = 4.465, p < 0.0001, Cohen’s d = 1.1428]. A similar reduction was observed in the FTD group relative to controls [t(49) = 2.861, p = 0.0296, Cohen’s d = 0.7982]. However, no significant differences were found between the FTD and AD groups in global averages [t(56) = 1.276, p = 0.3569]. This can be seen in Fig. 4.

**Fig. 5:**
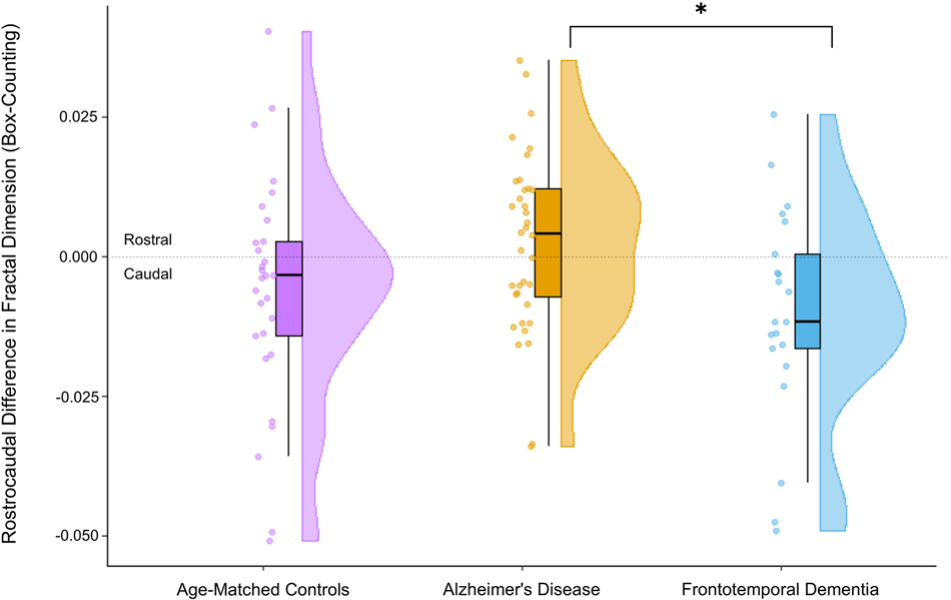
Raincloud plot comparing the rostrocaudal difference between AD, FTD, and age-matched controls. Significant differences are noted with * (p < 0.05). Negative values indicate caudal dominance and positive values indicate rostral dominance. On the left of each entry are the raw data points. In the middle is a boxplot. On the right is the point density plot. A single outlier was removed from the FTD group and the significant difference between AD and FTD retained.

A one-way ANOVA revealed significant group differences in rostrocaudal fractal dimension values [F(2, 84) = 4.426, p = 0.0149, r² = 0.0953]. Post-hoc comparisons using Tukey’s HSD confirmed indicated significant differences between the AD and FTD groups [t(56) = 3.009, p = 0.0120, Cohen’s d = 0.7756], where FTD exhibited a caudal dominance, while AD displayed a rostral dominance. This pattern remained even after controlling for an outlier (−0.0762) in the FTD group [t(55) = 2.658, p = 0.0395, Cohen’s d = 0.7105] (Fig. 5). No significant differences were found between the FTD group and age-matched controls [t(49) = 1.191, p = 0.3831], the difference between AD and age-matched controls was also not significant [t(63) = 1.808, p = 0.2218].

**Fig. 6:**
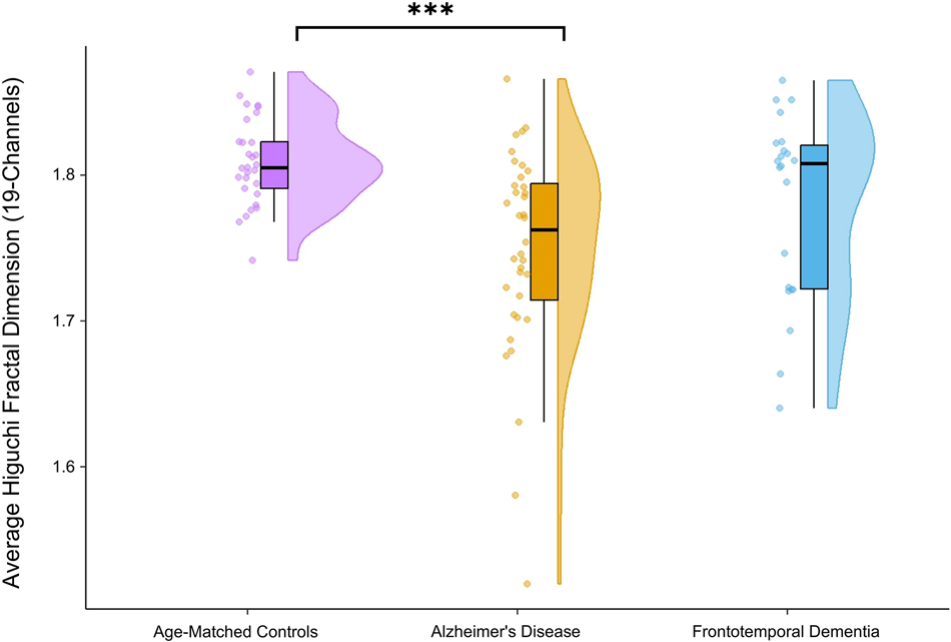
Raincloud plot comparing the 19-channel average Higuchi Fractal Dimension values between AD, FTD, and age-matched controls. Significant differences are noted with *** (p < 0.001). On the left of each entry are the raw data points. In the middle is a boxplot which includes the interquartile range, and the data point range represented by vertical lines above and below the interquartile range. On the right is the point density plot represented as a half violin.

To validate and expand upon our initial fractal dimension findings, we computed the Higuchi Fractal Dimension (HFD) across all 19 channels. ANOVA revealed significant group differences in global HFD values [F(2,84) = 8.601, p = 0.0004, r² = 0.1700], with AD patients showing significantly reduced HFD compared to controls [t(63) = 4.281, p = 0.0002, Cohen’s d = 1.1118]. No significant differences were found between AD and FTD or FTD and controls (Fig. 6).

### Reduced LRTCs in AD and FTD as Assessed by DFA

**Fig. 7:**
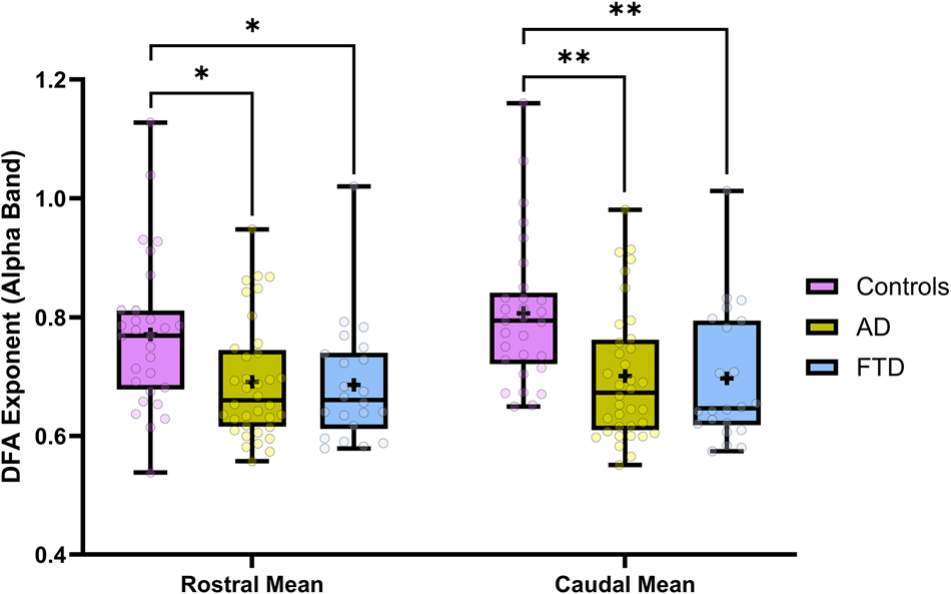
Boxplots representing the rostral (left) and caudal (right) means for LRTCs as assessed using DFA exponents for the alpha band (8 Hz to 13 Hz). Plotted is the interquartile range, “+” symbols represent the mean, and whiskers represent the data range, within each entry is the raw data represented as transparent dots. Significant differences are noted with * and ** (p < 0.05 and p < 0.01, respectively).

A one-way ANOVA revealed significant group differences in long-range temporal correlations (LRTCs) for both rostral [F(2, 84) = 5.371, p = 0.0064, r² = 0.1134] and caudal means [F(2, 84) = 8.338, p = 0.0005, r² = 0.1656] (Fig. 7). Post-hoc comparisons using Tukey’s HSD showed that within the rostral mean, no significant differences were found between AD and FTD. However, both groups differed significantly from age-matched controls, with AD [t(63) = 2.834, p = 0.0127, Cohen’s d = 0.6973] and FTD [t(49) = 2.577, p = 0.0205, Cohen’s d = 0.7401] displaying altered LRTC patterns. For the caudal mean, a similar pattern emerged, with both AD [t(63) = 3.631, p = 0.0012, Cohen’s d = 0.9019] and FTD [t(49) = 3.289, p = 0.0034, Cohen’s d = 0.9367] showing significant reductions compared to controls. Notably, the effect sizes for caudal differences were larger than those observed in the rostral mean, indicating more pronounced disruptions in caudal LRTC dynamics.

**Fig. 8:**
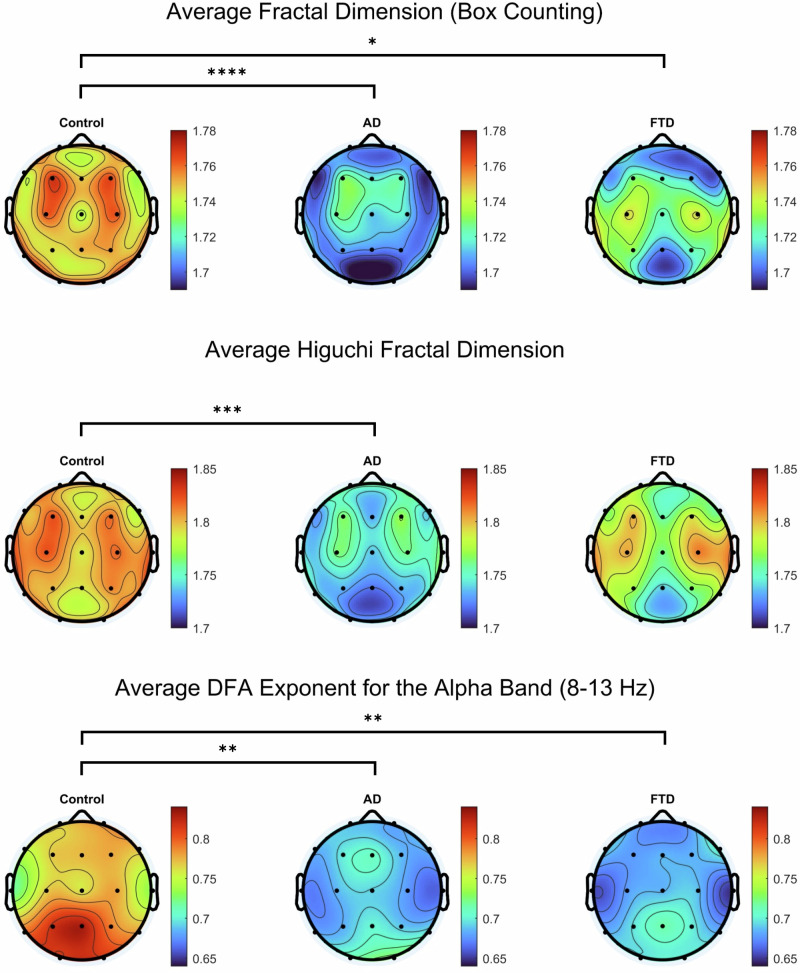
Topographic mapping of average fractal dimension values (top), average Higuchi Fractal Dimension (middle), and average DFA exponent of the alpha band (bottom). Black dots represent each of the 19 channels and are arranged according to the 10–20 system. Significance bars indicate a difference in the 19-channel average. Significant differences are noted with *, **, ***, and **** (p < 0.05, p < 0.01, p < 0.001, and p < 0.0001, respectively).

To further investigate alterations in EEG complexity across groups, we analyzed fractal dimension, both box counting and HFD, and DFA exponents at the scalp level (Fig. 8). Both measures revealed significant reductions in AD and FTD compared to controls, with the most pronounced decreases observed in AD. Topographic maps of fractal dimension show widespread reductions across the scalp in AD, while FTD exhibits a more regionally confined decline. Similarly, DFA exponents for the alpha band (8–13 Hz) were significantly lower in both neurodegenerative groups, suggesting a breakdown in long-range temporal correlations. These findings reinforce the notion that AD and FTD are characterized by progressive reductions in EEG signal complexity.

**Fig. 9:**
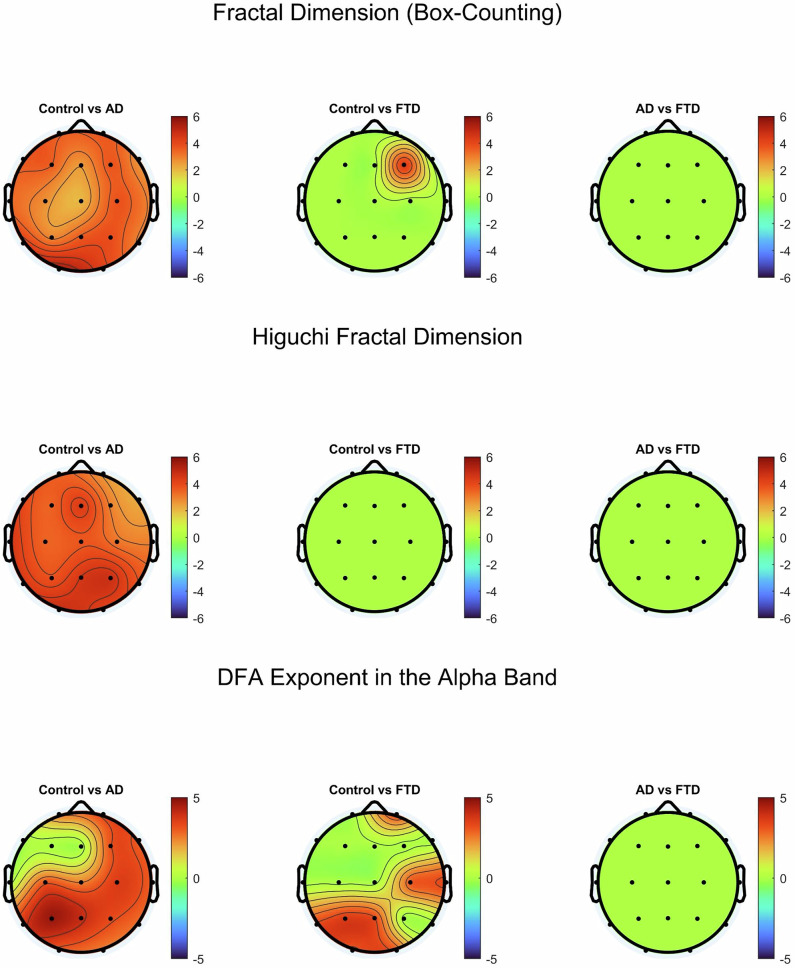
Topographic plots of the t-value comparisons with channels that passed the False discovery rate (FDR) correction in complexity measures: Box-counting, HFD, and DFA in the alpha band. Black dots represent each of the 19 channels and are arranged according to the 10–20 system. Color bars represent the t-values. The leftmost column compares controls to AD, the middle column compares controls to FTD, and the rightmost column compares AD to FTD.

Finally, to assess spatial specificity of group differences in signal complexity, we also generated topographic t-value maps using the FDR correction for fractal dimension (box-counting), HFD, and DFA exponents in the alpha band (Fig. 9).

### Results from dataset 2

**Fig. 10:**
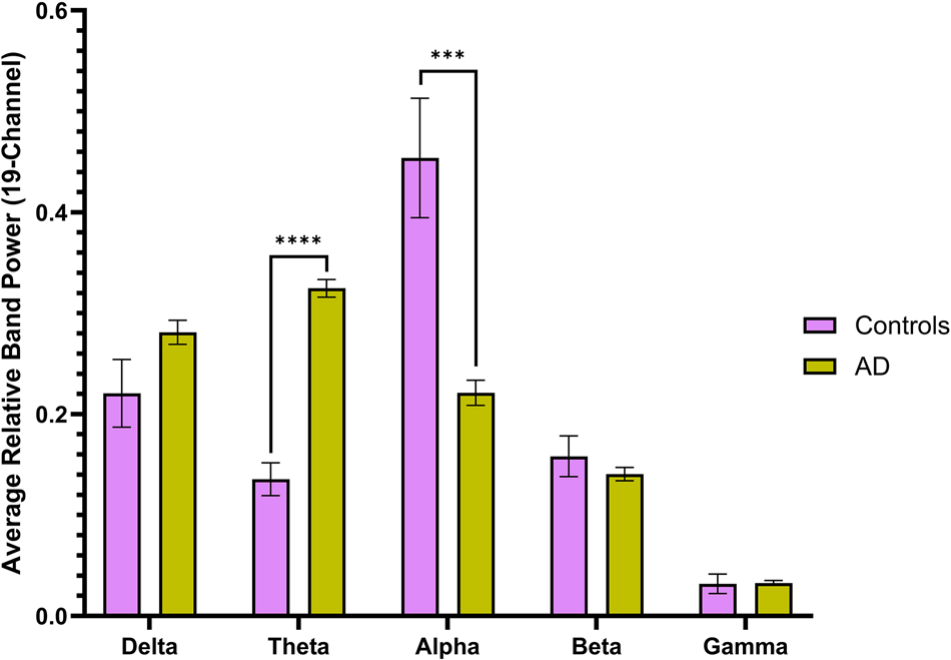
Comparison of the 19-channel average of relative power values across 5 frequency bands: delta (1–4 Hz), theta (4–8 Hz), alpha (8–13 Hz), beta (13–30 Hz), and gamma (30–45 Hz) for dataset 2. Significant differences are noted with *** and **** (p < 0.001, and p < 0.0001, respectively). Error bars represent standard error of the mean.

**Fig. 11:**
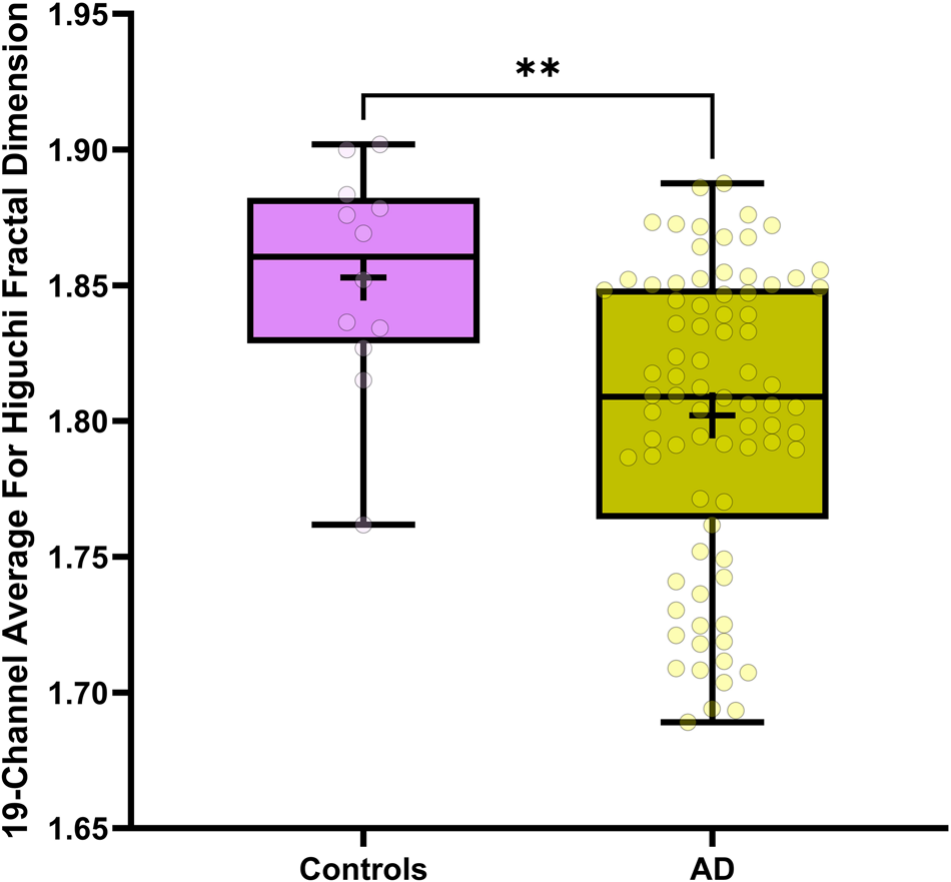
Boxplot comparison of HFD values (19-channel average) between control and HFD for dataset 2. Plotted is the interquartile range, “+” symbols represent the mean, and whiskers represent the data range, within each entry is the raw data represented as transparent dots. Significant differences are noted with ** (p < 0.01).

To evaluate the robustness and external validity of our findings, we applied the same signal processing and analytical pipeline to an independent open-source EEG dataset. This second dataset included a substantially larger sample of probable AD patients but fewer age-matched healthy controls relative to the primary dataset. Our goal was to determine whether key group-level differences in spectral power and signal complexity observed in the original analysis would replicate under different sampling conditions and acquisition parameters. When observing the 19-channel averages for relative band power following the false discovery rate (FDR) correction for multiple comparisons, AD displayed significantly higher theta power compared to controls [U = 19, p < 0.0001, Cohen’s d = 2.7453] with delta nearing significance [U = 311, p = 0.0504, Cohen’s d = 0.5417], whereas alpha values were significantly lower [U = 183, p = 0.0004 Cohen’s d = 1.4084] (Fig. 10). No significant differences were observed in beta or gamma bands. When observing the 19-channel averages in fractal dimension, significance was only observed with HFD (kmax=32) wherein AD displayed significantly lower values than controls [t(90) = 3.094, p = 0.0026, Cohen’s d = 1.0608] (Fig. 11).

## Discussion

To investigate EEG signatures of neurodegenerative disorders, specifically AD and FTD, we applied multiple signal processing techniques, including power-based analysis and complexity measures such as fractal dimension and DFA. We focused on broad changes (frontal, caudal, and global), and were able to observe significant differences between AD and FTD using box-counting fractal dimension measures. The differentiation of AD and FTD from controls were observed most prominently from changes in slow frequencies and fractal dimension. The purpose of the inclusion of signal complexity measures was to highlight the power of novel analysis techniques in signal processing when applied to disorders. Scalp recordings can reflect underlying neuronal dynamics^5^, given the altered microstructure in neurodegenerative disorders, these alterations may reflect in scalp recordings. Consistent with prior work^22–27^, we found that the HFD reliably differentiated AD from control participants. Importantly, this method complements our findings from the traditional box-counting approach, which identified a rostrocaudal asymmetry between AD and FTD. While HFD did not differentiate AD from FTD, its ability to detect global complexity reductions reinforces its value for characterizing neurophysiological decline. While HFD appears more sensitive to global signal breakdown, box-counting may better capture spatial distribution of complexity. This dual-method approach reinforces the value of signal complexity as a biomarker and highlights the need for continued methodological refinement in EEG-based dementia research.

Using multiple techniques can give numerous insights on the signal based features of a given disorder, and potentially be consistent with underlying symptoms. In neurodegenerative disorders, not only is power in the alpha band reduced broadly, but also reduced is signal complexity in both short, as assessed by fractal dimension, and large time scales, as assessed using DFA. The rostrocaudal difference in signal complexity is consistent with literature on fractal dimension analysis of AD patients wherein there is reduced caudal complexity^22,23^. This indicates that not only were the information content and space-filling properties of the signal reduced, but also that the distribution of information along the rostrocaudal axis was abnormally altered in AD. While band power analysis provides valuable data, signal complexity measures offer an additional effective approach for identifying neurodegenerative disorders. The reduction in relative alpha power was also accompanied by increases in the relative power of the slower frequencies, delta and theta. This compensatory change may reflect disturbances in the excitatory-inhibitory gradient, further supplemented by reported roles of alpha in cyclic inhibition^12^. Alpha activity is proposed to arise from the slow conduction speeds observed in the majority of axons in the brain^30^. Disruptions in alpha rhythms may be linked to disturbances in the excitation-inhibition gradient characteristic of neurodegenerative disorders^12^. Excess amyloid beta deposits interact with the glutamatergic system and can impact neuronal glutamate release^31^, while hyperphosphorylated tau, a hallmark of FTD, has also been associated with increased glutamate release in transgenic mouse models^32,33^. Neuronal activity follows lognormal distributions, with most neurons exhibiting low firing rates^31^. Additionally, the predominance of slow-conducting neurons, due to thin myelination, is thought to contribute to slow-frequency dominance in EEG signals^4,30^. In AD, myelin alterations^34^ may further disrupt conduction speed, potentially contributing to alpha rhythm abnormalities. Given these structural and functional changes, EEG techniques may capture disturbances in the excitation-inhibition gradient beyond traditional band power analysis. Specifically, reductions in conduction speed due to myelin degradation could potentially underlie the increased relative power in lower-frequency activity observed in scalp recordings.

What differentiates complex activity such as that which is observed in biological rhythms, from random signal is 1/f-like behavior in various domains, such as between frequency and power or amplitude fluctuations and time in log-log space^4^. From analysis of LRTCs, scaling behaviors between amplitude fluctuations over multiple scales of time can be observed. The results from this research indicate that while LRTCs are present in AD and FTD, they more closely resemble LRTCs seen in a random signal such as that of white noise than would be observed in healthy age-matched controls. A similar decay in scaling behaviors can be observed even in healthy individuals under sleep deprivation^35^. Most research on LRTCs is conducted with slow frequencies within the alpha and beta range, given the changes in higher frequencies (>40 Hz) that can occur in AD^36^, more research is needed on the long-range spatiotemporal properties of high-frequency oscillations in neurodegenerative disorders. This can become difficult as muscle artifact contamination gradually increases after the 30 Hz range^37^, but with a proper recording setup and preprocessing pipeline it is possible.

To address concerns regarding generalizability, we conducted a post hoc validation of our primary findings using a second open-source EEG dataset. While this secondary dataset featured a substantially larger cohort of probable AD patients, it included fewer age-matched controls and shorter recording durations. Despite these limitations, the replication of key findings, specifically the increase in theta power and the reduction in alpha power and signal complexity in AD, suggests that the observed EEG alterations are not specific to a single dataset or acquisition protocol. Notably, HFD again proved sensitive to complexity reductions in AD, reinforcing its utility as a candidate biomarker. However, the abbreviated recording length precluded DFA analysis, limiting the scope of comparison. These results highlite the need for future studies utilizing multi-site datasets with standardized protocols to further assess the diagnostic utility of EEG complexity measures in diverse clinical populations.

While our findings demonstrate meaningful group-level differences in EEG signal complexity and spectral power, several limitations warrant consideration. Most notably, the sample size, though comparable to other open-access EEG datasets, limits the generalizability of these results. Larger, multi-site datasets will be essential for validating the reproducibility and diagnostic utility of these measures across diverse populations. Additionally, while the current analysis focused on resting-state EEG, future work should explore task-related dynamics and longitudinal data to better capture disease progression and individual variability.

Differentiating neurodegenerative disorders based on electrophysiological profiles remains a critical challenge in neuroscience. This study demonstrates that EEG-based complexity measures, particularly fractal dimension and long-range temporal correlations, can capture disease-related alterations in neural dynamics beyond what is detectable through spectral analysis alone. Notably, the identification of a rostrocaudal asymmetry in complexity measures differentiating AD and FTD represents a novel finding with potential diagnostic relevance. By integrating traditional and emerging EEG metrics, we provide a multiparametric framework for evaluating large-scale brain dysfunction in dementia. These approaches, while preliminary, suggest that EEG complexity may serve as a sensitive indicator of underlying network disruption.

As complexity-based techniques continue to evolve, their combination with machine learning and multi-modal datasets could enhance classification accuracy and clinical translation. Although EEG lacks the spatial resolution of MRI or PET, its millisecond temporal sensitivity and scalability make it well-suited for identifying dynamic biomarkers of neurodegeneration. With further validation in larger and longitudinal cohorts, EEG may play an increasingly important role in accessible, non-invasive monitoring of disease progression.

## Methods

### Data acquisition, subjects, and recording from Miltiadous et al. (2023)

Data was acquired from the OpenNeuro EEG database, we used recordings uploaded to the database by Miltiadous et al. (2023). This dataset consisted of resting state eyes closed EEG recordings from Alzheimer’s Disease (AD) patients (n = 36), Frontotemporal dementia (FTD) patients (n = 23), and age-matched healthy controls (n = 29). The mean age for the AD group was 66.4 (SD = 7.9), for the FTD group the mean age was 63.6 (SD = 8.2), and a mean age of 67.9 (SD = 5.4) for the control group. The dataset was balanced by gender with n = 44 females and n = 44 males (12 males in the AD group, 18 in the control group, and 14 in the FTD group). Also included in the study were Mini Mental Status Examination (MMSE) scores which range from 0 to 30 and are indicative of cognitive decline. The mean MMSE score in the AD group was 17.75 (SD = 4.5), 22.17 (SD = 8.22) in the FTD group, and all MMSE scores in the healthy control group were 30. The data was recorded by Miltiadous et al. (2023) using the Nihon Kohden EEG 2100 device, using a standard 19-channel cap with the 10–20 international system for electrode placement. For reference electrodes the researchers utilized A1 and A2 mastoid electrodes. The sampling rate for recording was 500 Hz. The high cutoff frequency for recording was 70 Hz^38^.

### EEG preprocessing

Miltiadous et al. (2023) filtered the data between 0.5 Hz and 45 Hz using a butterworth filter. Within the EEGLAB toolbox for MATLAB they then performed artifact subspace reconstruction for large artifact removal, which is a technique for removing artifact laden time points. Additional eye and muscle artifacts were removed using independent component analysis (ICA), following this, components were labeled with the ICLabel function, with eye and muscle components being automatically removed. With the processed data from Miltiadous et al. (2023), we performed extra steps in that the data was re-filtered from 1 Hz to 45 Hz to remove low frequency noise. We then re-referenced the data to the common average. After this, we epoched 15 s of artifact free data and utilized it for band power and fractal dimension computations. In the FTD group, 1 recording was excluded for excess artifacts.

### Fractal dimension analysis

Fractal dimension values were obtained using the myFractal plugin for EEGLAB created by Saab & Shaw (2016). This plugin utilizes a time-series box counting method to compute the average fractal dimension values for each channel^39^. Fractal dimension computation was performed on the broadband signal (1–45 Hz). For this analysis we took the 19-channel global average in fractal dimension, the rostral channel average (Fp1, Fp2, F7, F3, Fz, F4, and F8), caudal channel average (T5, P3, Pz, P4, T6, O1, and O2), and the rostrocaudal difference. The rostrocaudal difference was defined as the caudal mean subtracted from the rostral mean wherein positive values indicate caudal dominance and negative values indicate caudal dominance.

### Relative band power

For obtaining relative band power values we utilized the Darbeliai extension for EEGLAB. The fast fourier transform window length was set to 2 s, and the spectrum steps was set to 1 Hz. The frequency bands for the computation included delta (1–4 Hz), theta (4–8 Hz), alpha (8–13 Hz), beta (13–30 Hz), and gamma (30–45 Hz). The absolute band power was divided by the broadband absolute power to obtain relative band power values, expressed as a ratio. Similar to section “Fractal Dimension Analysis”, the 19-channel average, rostral average, caudal average, and rostrocaudal difference were calculated.

### Long-range temporal correlations

**Fig. 12:**
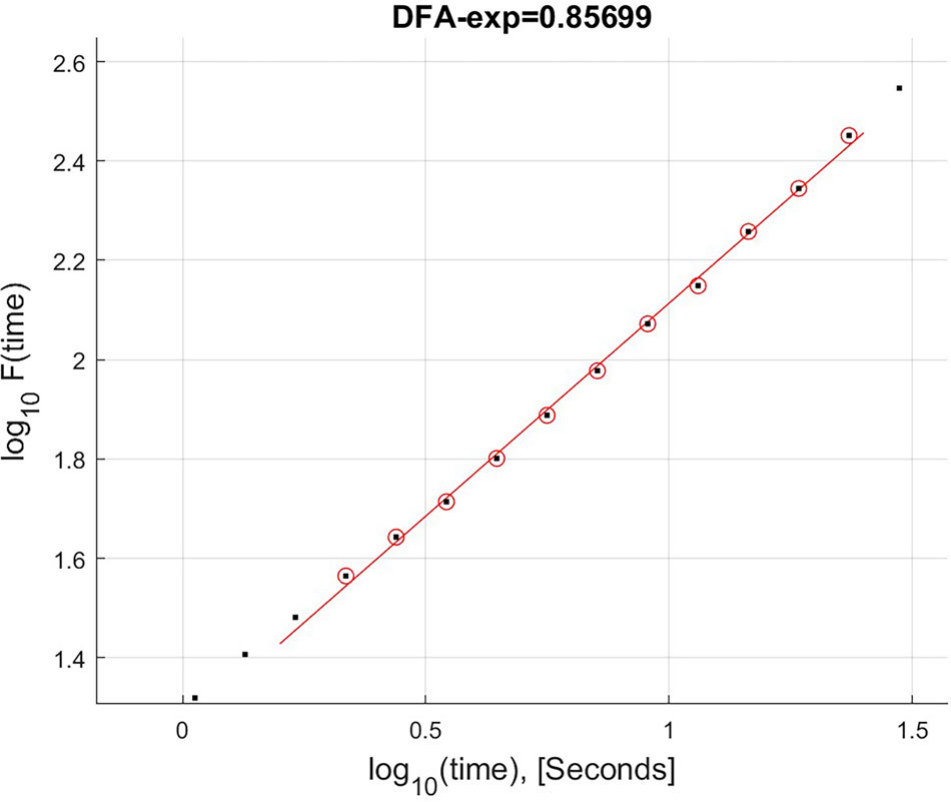
Example of a scaling relationship between amplitude fluctuation of the alpha band on the y-axis and time scales on the x-axis from a single age-matched control participant in the Pz channel, plotted in log-log space.

LRTCs were computed with the detrended fluctuation analysis (DFA) method. First, 300 s of data was utilized for DFA, which was computed using the Neurophysiological Biomarker Toolbox (NBT) for MATLAB. First, narrowband filtering was performed using a finite impulse response filter, with the high pass filter set to 8 Hz and the low pass set to 13 Hz (alpha band). Amplitude envelopes were then extracted from the data using a Hilbert transform, from which the DFA exponent was computed for all 19 channels with a fitting interval between 2 and 25 s, a calculation interval between 0.8 and 30 s, the window overlap set to 50%, and the number of windows set to 10. The DFA command in the NBT computes the mean signal, detrends the signal, computes the root mean square, and performs power law fitting. Rostral and caudal averages were then computed from their respective channels as mentioned in section “Fractal Dimension Analysis”. An example of a relationship between fluctuations in amplitude and the time scales can be observed in Fig. 12. DFA exponent values closer to the range of 0.8 to 1 indicate greater LRTC strength similar to what can be seen with pink noise, whereas in values near 0.5, LRTCs are present but are weaker which is more similar to white noise.

### Higuchi fractal dimension computation

**Fig. 13:**
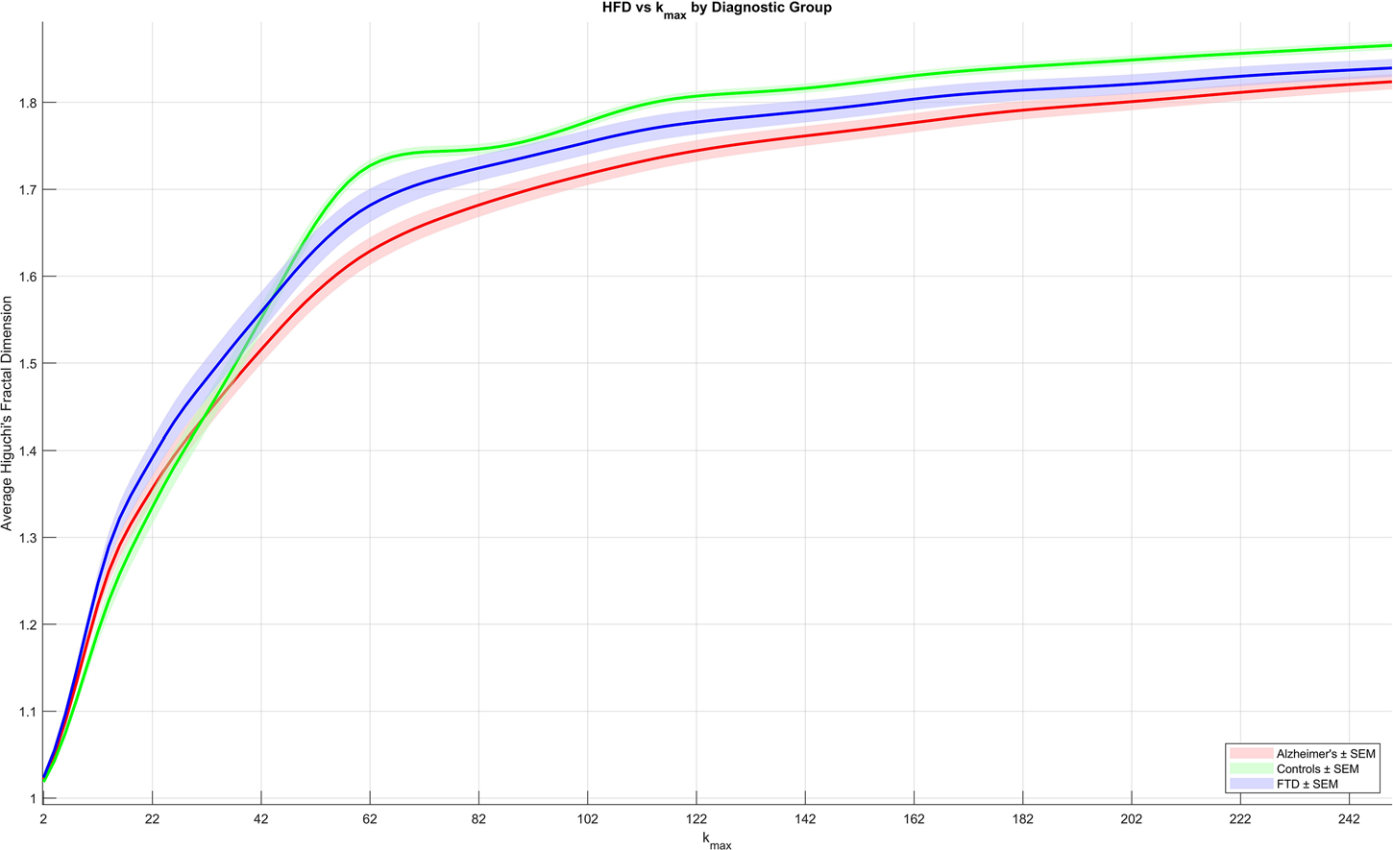
The 19-channel average for HFD values across kmax values tested between 2 and 250. 126 was defined as the kmax value to be used in this analysis as it corresponded to the median HFD value across all subjects. Solid lines indicate the mean whereas colored shaded regions surrounding the solid line indicate the standard error of the mean (SEM). Controls, AD patients, and FTD patients are denoted by the colors green, red, and blue, respectively.

The Higuchi Fractal Dimension (HFD) was computed using an open source code provided by Selvam (2025) which integrates Higuchi’s algorithm^40,41^. Fractal properties are assessed using curve lengths of various step sizes. The defined parameter, kmax, refers to the maximum length of data points in a time series for the assessment of HFD and varies by case^42^. We tested kmax values ranging from 2 to the Nyquist limit of our dataset (i.e., 2, … 250) between groups (Fig. 13). HFD for the 19-channel average at each kmax value for each subject was computed. HFD values from non-overlapping windows of 1 s were averaged for each epoch. We selected a kmax value of 126 for this analysis as it corresponded to the median HFD value across all subjects, consistent with other experiments utilizing this technique^22^.

### Dataset 2 description

To validate the results from the Miltiadous et al. (2023) dataset we utilized a second open-source dataset from Vicchietti et al. (2023). This dataset consisted of 19-channel recordings under the 10–20 electrode placement system. Participants include 12 healthy elderly controls with their eyes closed, 12 controls with their eyes open, 80 probable AD patients with their eyes closed, and 80 probable AD patients with their eyes open, wherein 8 s of data was provided for each. The 160 probable AD patients diagnosed through the National Institute of Neurological and Communicative Disorders and Stroke and the Alzheimer’s Disease and Related Disorders Association, and Diagnostic and Statistical Manual of Mental Disorders-III-R criteria^43,44^. The data was sampled at 128 Hz, preprocessing steps employed by us were similar to those described in section “Data Acquisition, Subjects, and Recording from Miltiadous et al. (2023)” with the exception of ASR. DFA was not performed on this dataset due to the recording length. The HFD parameter, kmax, was obtained using the methods described in the previous section and we used a kmax of 32 for this analysis.

### Statistical analysis

Statistics were performed using the GraphPad Prism 10.4.0 software, except for the multivariate analysis of variance (MANOVA) in section “Fractal Dimension and Rostrocaudal Differences Between AD and FTD”, which was performed using IBM SPSS 29.0 software. Data normality was observed using D’Agostino-Pearson tests prior to comparison. Comparisons between conditions were performed using Tukey’s honestly significant difference (HSD). Group analysis was performed using one-way analysis of variance (ANOVA). Effect sizes were calculated using Cohen’s d wherein the mean difference is divided by the pooled variance, with a threshold of Cohen’s d = 0.5. Single channels were not compared between groups but rather the averages of broad regions: 19-channel average, rostral mean, caudal mean, and the asymmetry (difference) between the rostral and caudal sensors. Plotting functions were performed using RStudio and GraphPad Prism for data visualization, whereas topographic EEG plots were generated using a custom MATLAB script for the averages of each channel per condition and t-value comparisons between groups.

## Data Availability

The EEG data used in this study are openly available through the OpenNeuro repository. Specifically, the dataset from Miltiadous et al. (2023) [ds004504] was used. All preprocessing scripts and analysis code used in this study are available upon reasonable request.
